# Endovascular Management of a Deep Femoral Artery Perforating Branch Pseudoaneurysm after Total Hip Arthroplasty: A Case Study

**DOI:** 10.1155/2022/5925839

**Published:** 2022-11-23

**Authors:** Sami Nabhani, Eric Cheysson, Pamela Sabbah, Georges Baaklini

**Affiliations:** ^1^Division of Vascular Surgery, Saint Georges Hospital-University Medical Center, Ashrafieh, Beirut, Lebanon; ^2^Division of Vascular Surgery, Centre Hospitalier de Pontoise, Pontoise, France

## Abstract

Pseudoaneurysm of the profundal femoris artery (PFA) following total hip arthroplasty (THA) is a rare and unusual complication. Awareness of this complication and a high level of suspicion allow early diagnosis and treatment, thereby reducing the morbidity of this condition. We present a case of a pseudoaneurysm of a perforating branch of the PFA after revision THA which was treated successfully by selective coil embolization.

## 1. Introduction 

Pseudoaneurysm of the PFA or its branches is an unusual and serious complication after THA. Vascular injuries are a rare complication after orthopedic surgery of the hip. The complication rate is reported to be approximately 0.2–0.3% [1–3]. In a large series of >13,000 cases, an incidence of 0.04% in primary THA increasing to 0.19% in revision surgery was seen [4]. Such complications account for the third most common iatrogenic injury after those caused by catheterization and trauma [1]. They can result in significant mortality (7%) [5], amputation (1.6%), and persistent ischemia (7.3%) [4]. These complications can include thromboembolism, direct vessel laceration, pseudoaneurysm, and arteriovenous fistula formation. Etiologies are direct trauma from surgical instruments, joint manipulation and dislocation, protrusion of cement or screws, excessive retraction, and heat injury from the exothermic reaction generated during cement (methyl methacrylate) polymerization [1, 3, 6–8].

Furthermore, there is an increased incidence in patients undergoing redo arthroplasty [9]. As stated by Del Prince et al, the prevalence of vascular injuries post redo arthroplasty ranged between 0.06 and 0.2%. These injuries are classified into 2 groups: acute-onset vascular injury and delayed-onset vascular injury. Acute onset vascular injury may occur intraoperatively secondary to direct trauma from surgical instruments or cement. As mentioned by An et al., delayed vascular injury can result from postoperative trauma to vessels [10, 11].

Vascular damage resulting from this procedure may present in many ways including pain, obvious bleeding, hemodynamic instability, anemia, a pulsatile mass, and limb ischemia [12]. De Boer et al. stated that the most common locations of pseudoaneurysms after THA are the external iliac artery and the common femoral artery [13]. Injuries and pseudoaneurysms of the PFA and its branches are very rare after THA as the artery is not close to the operation site [12, 14]. Some cases resulting from chronic repeated trauma by a projecting screw were reported after intertrochanteric fracture treated by internal fixation with a dynamic hip screw plate [15, 16].

Recent studies have concentrated on a pseudoaneurysm of the PFA that has been reported approximately two months after THA, in contact with a cement leak from the hole of the removed screw from the prior osteosynthesis of an intertrochanteric fracture [17, 18]. Öztürk et al. reported a pseudoaneurysm of the PFA after bipolar hemiarthroplasty, caused by the sharp edge of a fractured lesser trochanter [14].

In light of this, a risk factor for redo procedures is the presence of vascular damage due to THA, an uncommon complication for these injuries [2]. Because of its location deep in the thigh, pseudoaneurysm of the PFA often presents late with persistent pain and swelling. Patients may not present with the usual sign of a pulsatile mass [19]. Diagnosis of pseudoaneurysm is especially dependent upon suspicion because the symptoms and signs are nonspecific which makes it a challenging process [4]. As a result, if there is persistent pain and swelling in the thigh after THA, additional vascular investigations should be performed. Noting that, it can be diagnosed by Doppler ultrasonography or computed tomography. Angiography, however, is needed for definitive diagnosis and for endovascular repair if needed.

In this article, the authors present a case of a pseudoaneurysm of a perforating branch of the PFA following revision THA treated successfully by selective coil embolization.

## 2. Materials and Methods

This article presents a case study of a 50-year-old lady with no significant medical history who underwent a right THA for congenital hip dysplasia. Recurrent dislocations were the indication for revision THA after 6 years.

**Figure 1:**
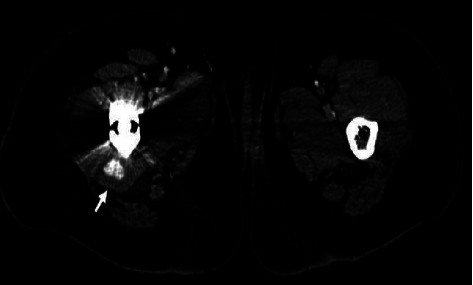
CT with contrast showing the right deep femoral artery pseudoaneurysm.

**Figure 2:**
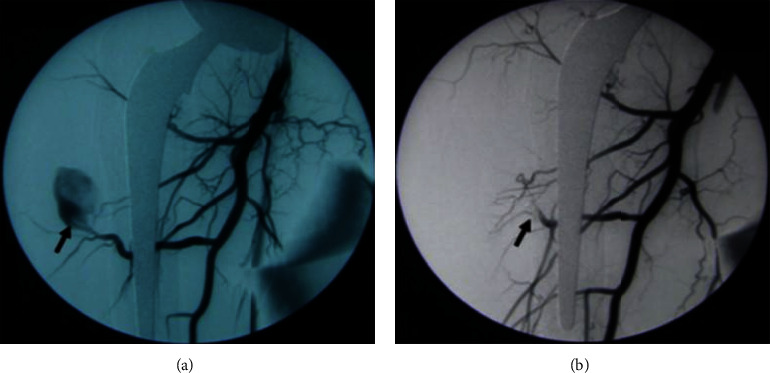
(a) Arteriography showing the pseudoaneurysm pre-embolization. (b) Arteriography showing resolution of the pseudoaneurysm postembolization.

**Figure 3:**
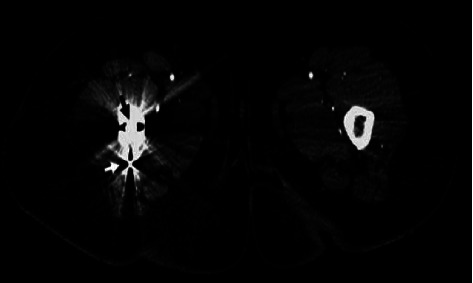
CT with contrast showing resolution of the pseudoaneurysm after embolization (done after 6 months).

Two weeks postoperatively, during her stay at the rehabilitation center, she started complaining of severe, worsening pain localized to the right groin. Clinical neurologic and vascular examinations were normal. Radiographs showed satisfactory positioning of the hip prosthesis. The orthopedic consultation revealed no orthopedic origin of her complaints. Duplex ultrasound showed a small pseudoaneurysm arising from the profunda femoris (ying-yang pattern on the color flow and to-and-fro waveform on the spectral doppler); no signs of venous thromboembolism were noted. A trial of ultrasound-guided compression was done without success. Computed tomography (CT) scan revealed a 14 × 22 mm pseudoaneurysm originating from a distal perforating branch of the right PFA (Figure 1). Considering that no thrombin was available for an ultrasound-guided injection of the pseudoaneurysm, the endovascular repair was our treatment of choice. Arteriography was done via left femoral artery retrograde access confirming the presence of the pseudoaneurysm (Figure 2(a)). Subsequently, selective catheterization of the distal perforating branch of the right PFA was performed using Progreat™ microcatheter (Terumo Corporation, Tokyo, Japan) and successful embolization was achieved using 2 and 3 mm platinum coils (Balt Extrusion, Montmorency, France), placed distal and proximal to the neck of the pseudoaneurysm. Subsequent injection of the contrast medium demonstrated no flow into the pseudoaneurysm (Figure 2(b)). Following embolization, the patient had a gradual improvement of her symptoms. The patient revealed that she had no pain, claudication, or swelling at follow-up 6 weeks later, and the control CT scan showed no recurrent vascular lesion over a 6-month period (Figure 3).

## 3. Results and Discussion

In our case, the pseudoaneurysm originated from a distal perforating branch of the PFA. There was no evidence of direct trauma from surgical instruments because it was far from the operation site; also, the injured artery was not in contact with a protrusion of cement, bone edge, or screws. During the operation on a redo case, the hip was moved out of place, which could have torn the blood vessel.

Moreover, the presentation was a nonspecific groin pain 15 days postoperatively, which raised the suspicion of vascular complications and indicated a diagnostic procedure.

Doppler ultrasonography and a CT scan can be used to diagnose pseudoaneurysms in these cases. Angiography, however, was needed for a definitive diagnosis and transcatheter treatment.

Open surgical repair of a pseudoaneurysm of the PFA can be difficult because of the location of the PFA, deep in the muscle compartment of the thigh. Transcatheter or ultrasound-guided percutaneous injection of thrombin, coil, or fibrin adhesive has been reported to occlude a similar pseudoaneurysm [20–22]. Transcatheter embolization with a coil provided an easier and less invasive technique than open surgical repair in this case [15, 16].

Nozawa et al. reported a case of pseudoaneurysm of the PFA after THA was treated by transcatheter embolization of the PFA [23]. Lund et al. described the treatment of pseudoaneurysm of the median circumflex artery following THA by coil embolization using selective catheterization [24]. Zhang et al. described 4 cases of successful embolization procedures done to deep femoral artery pseudoaneurysms after hip fracture surgery [25].

Successful embolization in our case was achieved using selective catheterization of the perforating branch of the PFA and coil embolization distal and proximal to the neck of the pseudoaneurysm to insure complete exclusion. Some opted for endovascular repair using a covered stent. [26] However, covered stent placement to seal the pseudoaneurysm was not feasible in our case, as it originated from a distal branch. A US-guided thrombin injection was not available at the time of intervention, although it could also have been considered a treatment option.

## 4. Conclusion

Following THA, pseudoaneurysm of the PFA is quite rare. Early identification and treatment are made possible by knowledge of this consequence and a high degree of suspicion, particularly in redo surgeries. Since it is simpler to reach the deep femoral artery branches and less intrusive than open surgical repair, the endovascular repair was the authors' method of choice in this situation. This article presented a successful instance of selective coil embolization for the treatment of a pseudoaneurysm of a perforating branch of the PFA after revision THA. This technique was effective and will be useful in future situations similar to this.

## Data Availability

All references are provided in the submitted document.
